# Associations in the continuum of care for maternal, newborn and child health: a population-based study of 12 sub-Saharan Africa countries

**DOI:** 10.1186/s12889-016-3075-0

**Published:** 2016-05-17

**Authors:** Patrick Opiyo Owili, Miriam Adoyo Muga, Yiing-Jenq Chou, Yi-Hsin Elsa Hsu, Nicole Huang, Li-Yin Chien

**Affiliations:** International Health Program, Institute of Public Health, School of Medicine, National Yang-Ming University, Taipei, Taiwan; Institute of Community Health and Development, Great Lakes University of Kisumu, Kisumu, Kenya; Institute of Public Health, School of Medicine, National Yang-Ming University, Taipei, Taiwan; School of Health Care Administration, Taipei Medical University, Taipei, Taiwan; Institute of Hospital and Health Care Administration, National Yang-Ming University, Taipei, Taiwan; Institute of Clinical and Community Health Nursing, National Yang-Ming University, Taipei, Taiwan

**Keywords:** Continuum of care, Maternal, newborn and child health, Sub-Saharan Africa, Structural equation modeling

## Abstract

**Background:**

Despite the progress in the Millennium Development Goals (MDGs) 4 and 5, inequity in the utilization of maternal, newborn and child health (MNCH) care services still remain high in sub-Saharan Africa (SSA). The continuum of care for MNCH that recognizes a tight inter-relationship between maternal, newborn and child health at different time periods and location is key towards reducing inequity in health. In this study, we explored the distributions in the utilization MNCH services in 12 SSA countries and further investigated the associations in the continuum of care for MNCH.

**Methods:**

Using Demographic and Health Surveys data of 12 countries in SSA, structural equation modeling approach was employed to analyze the complex relationships in continuum of care for MNCH model. The Full Information Maximum Likelihood estimation procedure which account for the Missing at Random (MAR) and Missing Completely at Random (MCAR) assumptions was adopted in LISREL 8.80. The distribution of MNCH care utilization was presented before the estimated association in the continuum of care for MNCH model.

**Results:**

Some countries have a consistently low (Mali, Nigeria, DR Congo and Rwanda) or high (Namibia, Senegal, Gambia and Liberia) utilization in at least two levels of MNCH care. The path relationships in the continuum of care for MNCH from ‘adequate antenatal care’ to ‘adequate delivery care’ (0.32) and to ‘adequate child’s immunization’ (0.36); from ‘adequate delivery care’ to ‘adequate postnatal care’ (0.78) and to ‘adequate child’s immunization’ (0.15) were positively associated and statistically significant at *p* < 0.001. Only the path relationship from ‘adequate postnatal care’ to ‘adequate child’s immunization’ (−0.02) was negatively associated and significant at *p* < 0.001.

**Conclusions:**

In conclusion, utilization of each level of MNCH care is related to the next level of care, that is – antenatal care is associated with delivery care which is then associated with postnatal and subsequently with child’s immunization program. At the national level, identification of communities which are greatly contributing to overall disparity in health and a well laid out follow-up mechanism from pregnancy through to child’s immunization program could serve towards improving maternal and infant health outcomes and equity.

## Background

The worldwide maternal and child mortality have declined by about 45 and 49 %, respectively, since 1990–2013 [1, 2]. Despite the progress in the Millennium Development Goals (MDGs) 4 and 5, utilization of maternal, newborn and child health (MNCH) care services still remain highly unequal—mothers from poorer households, rural areas and who are less educated are more likely to underutilize health care services—posing a critical challenge in the post-MDGs. The Countdown to 2015 indicators for tracking progress towards MDGs 4 and 5 was founded by the World Health Organization (WHO) in 2005 due to the growing realization that health-related MDGs needed radical changes and effective strategies for major improvement in the health outcomes, and one of its principal recommendation was to highlight and emphasize the importance of equitable coverage of vital services across the continuum of care for MNCH and to improve on outcome measurement [3]. The continuum of care for MNCH recognizes that there is a tight inter-relationship between maternal, newborn and child health at different time periods and location [4, 5]. Therefore, the focus of this study will be the period during pregnancy, childbirth and the early years of the newborn’s life.

The sub-Saharan Africa (SSA) is the most affected region globally. It accounts for 62 % of the global maternal deaths, that is—the risk of maternal death is 1 in 160 mothers as compared to 1 in 3700 mothers in high-income countries. And for each maternal death, another 30 mothers suffers from pregnancy-related problems and complications [2, 6]. Moreover, the risk of a child’s death in SSA is the highest (92 deaths/1000 live births), and is estimated to be 15 times higher than that of the high-income countries [1]. Understanding utilization behavior of the vital services in the continuum of care for MNCH is therefore imperative not only towards reducing inequity in health but also towards designing and implementing better strategies so as to improve the general public health perspective. For this reason, we explored the relationships from one level of care to the next level in the continuum of care for MNCH.

Specific analytical approaches, such as structural equation modeling (SEM), are often used to model complex relationships such as the continuum of care for MNCH. Such tools enable a clear understanding of how different levels of care are related to one another by estimating the paths associations in the model. Policy actions can be well-defined depending on whether the goal is to address health outcomes improvement or to design and implement a system that makes continuation of care possible, which would become a challenge if the relationships in the pathway of care are not clearly understood. Therefore, we explored the distributions in the utilization MNCH services in 12 SSA countries and further investigated the associations in the continuum of care for MNCH.

## Methods

### Study site and sampling

We used the standard Demographic and Health Surveys (DHS) data of 12 countries in SSA that was collected between 2010 and 2014: DR Congo (*n* = 18,716), Gambia (*n* = 8088), Liberia (*n* = 7606), Mali (*n* = 10,326), Namibia (*n* = 5046), Nigeria (*n* = 31,482), Rwanda (*n* = 9002), Senegal (*n* = 6842), Sierra Leone (*n* = 11,938), Tanzania (*n* = 8023), Togo (*n* = 6979), and Zambia (*n* = 13,457). The analysis was restricted to women who gave birth in the last 5 years before the survey. The specifics on the sampling criteria, data collection and processing is found in the final report of each country, accessible from the DHS Program website [7].

### Conceptual framework

**Fig. 1:**
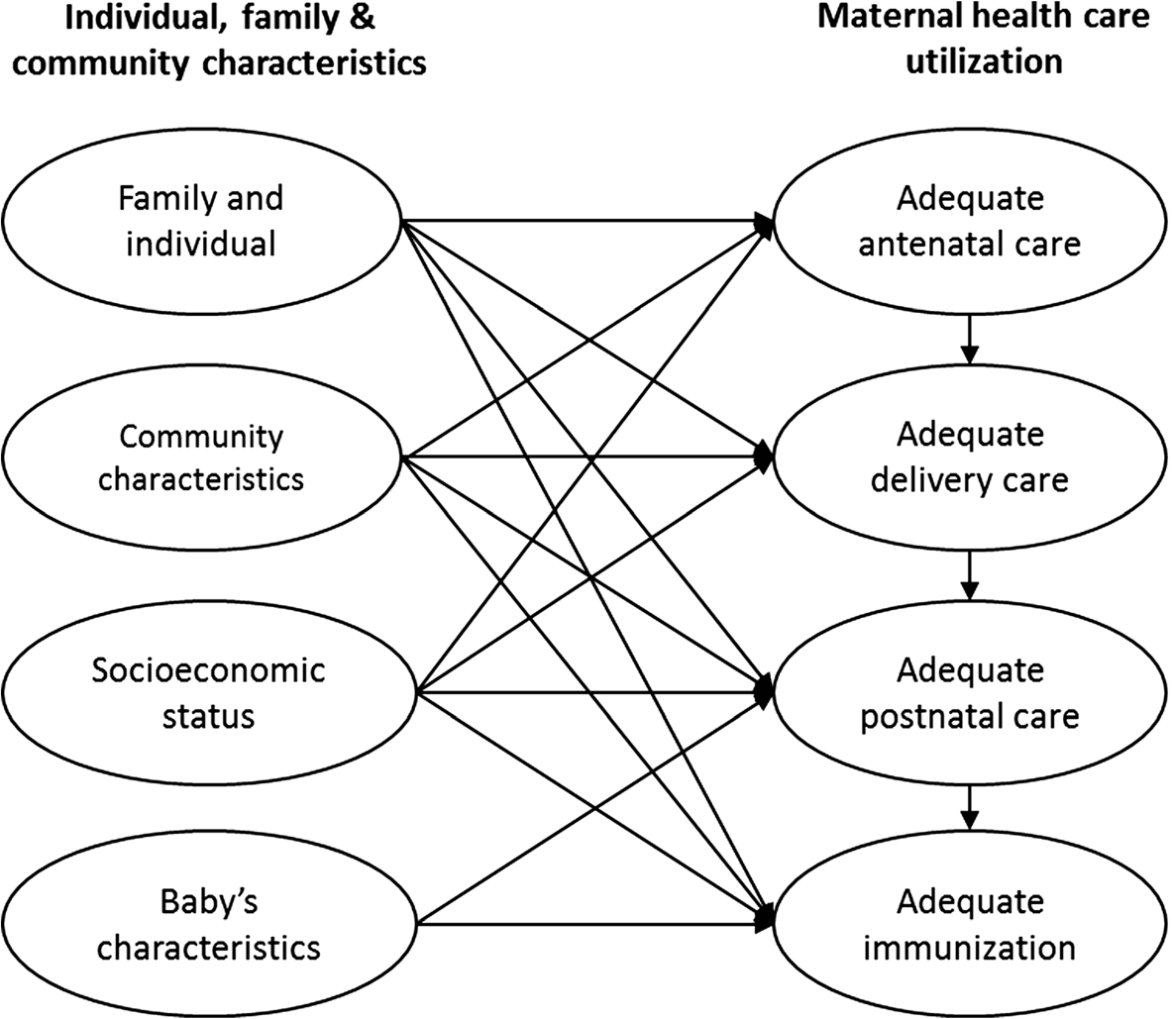
The conceptual model of the continuum of care for maternal, newborn and child health

The continuum of care for MNCH model was adopted from the WHO [8], though, we focused on the utilization of care in four levels: during pregnancy, at birth, postnatal care and infancy (Fig. 1). In the model, the continuum of care model is influenced directly by women’s, families’, communities’ and child’s characteristics. The model has 8 latent constructs that are related in different ways: adequate antenatal care; adequate delivery care; adequate postnatal care; adequate immunization; individual and family characteristics; community characteristics; socioeconomic status; and baby’s characteristics.

### Data and measures

#### Outcome variables

Several measurement indicators were used in this study to define the latent constructs (the codes in parenthesis were used for the generation of path diagram). The outcome indicators in the continuum of care for MNCH pathway were dichotomized, with the latent construct *child’s immunization program* (VACCINAT) measured using nine measurement variables measuring whether a child received the vaccine or not (1 = yes; 0 = otherwise) which included: four poliomyelitis vaccines (Polio0, Polio1, Polio2 and Polio3); Bacille de Calmette et Guérin vaccine (BCG); three diphtheria, pertussis and tetanus vaccines (DPT1, DPT2 and DPT3); and measles vaccine (measle). The latent construct *postnatal care* (POSTNAT) was measured using six indicators including received professional postnatal checkup (POSTCHEC), checked within 1 day after delivery (POSTIME), checked before discharge from the health facility (DISCHEC), newborn checkup (BABYPOST), newborn checked within 1 day after delivery (BABYTIME), and skilled newborn checkup (BABYPROF; skilled refer to a doctor, nurse, midwife or a clinical officer). On the other hand, the latent variable *delivery care* (DELIVERY) had only three indicators which included delivery at the health facility (FACDELV), skilled birth attendant (PROFDELV) and utilization of caesarean section services (CSECTION), while in measuring the latent construct *antenatal care* (ANTENAT) six indicators were used: received antenatal care (ANC), received four or more ANC (ANC4), initial ANC in the first trimester (ANCTRI), skilled ANC (ANCPROF), ANC at the health facility (ANCFAC), received six or more essential ANC services (ANCSERV). The ***essential ANC services*** included blood test, urine test, blood pressure check, received two or more tetanus injections, given malaria prophylaxis, given or bought iron tablets, and weight and height measured.

#### Independent variables

The independent determinants were classified into four latent variables as individual and family characteristics (PERSONAL), community characteristics (COMMUN), socioeconomic status (SES) and baby’s characteristics (BABY). The individual and family characteristics included marital status (MARITAL; single, married and formerly married), age group (AGEGRP; ≤ 20, 21–25, 26–30, 31–35, 36–40 and ≥ 40 years), birth order of the infant (BORD; 1st, 2nd, 3-4th and ≥ 5th born), household number (HHNO) and number of under-5 children (UNDER5NO). The community characteristics included country (COUNTRY), province (REGION) and whether the region is urban or rural (STRATA), while the SES latent construct included mother’s educational attainment (EDUCAT; no education, primary, secondary and higher), wealth index (WEALTH; poorest, poor middle, rich and richest), media exposure at least once a week—television, radio and newspaper (MEDIA; no exposure, exposure to any one, exposure to any two and exposure to all three), mother’s employment status (WOMJOB; not employed, professional, middle level job, agriculture and other), father’s employment (MANJOB; not employed, professional, middle level job, agriculture and other) and father’s educational attainment (MANEDUC; no education, primary, secondary and higher). The latent variable “baby’s characteristics” was measured using size of the newborn at birth (BABYSIZE), sex of the newborn (BABYSEX), and age in years (AGEYRS) and in months (AGEMTHS). The choice and classification of determinants of utilization of MNCH care was based on past studies [9–13]. Some of the indicators that were not collected in the survey of some countries (e.g. ethnicity in Tanzania and Rwanda, religion in Tanzania and health insurance in Rwanda) were not included in this study.

### Data analysis

Data analysis was performed in two stages using two statistical tools—the descriptive statistics using Stata version 13.0 and SEM analysis using Linier Structural Relationship (LISREL) version 8.80 [14, 15]. All analyses were weighted using complex survey method by incorporating the sampling strata, the cluster and the sampling weights to adjust for the sampling design. We used the Full Information Maximum Likelihood (FIML) in LISREL to estimate the SEM. The FIML is a modern approach that accounts for the Missing at Random (MAR) and Missing Completely at Random (MCAR) assumptions by using all the available information and the same pattern as if there were no missing data, with its results nearly similar to the multiple imputation procedure [16]. The covariances and means analyzed were estimated using Expectation Maximization (EM) procedure, which is only used to obtain starting values for the FIML procedure – the EM-algorithm can handle even up to 50 % of the missing data and generate reliable parameter estimates as if it were of a complete data. The final analysis revealed a pattern of 1200 missing-values, that is—11.92 % of missing values.

In the first stage of the analysis, data of each country were described in both proportions and graphically to observe the differences in utilization of care between the 12 countries. Data were then merged and utilization of MNCH care was computed across different countries, and the proportions within different characteristics were compared using *χ*^*2*^ test. The condition we used for statistical significance in this stage of analysis was α = 0.01. In the second stage, the SEM analysis using the FIML procedure was performed, which then produced overall parameter estimates of the 12 countries. Since the criteria for selecting a model vary between problems and discipline, we followed the method used in SEM which includes five basic steps—model specification, identification, estimation, testing and modification—that had been described elsewhere in detail [17, 18]. All theoretically identified measurements and determinants are included in the model specification step; while a set of distinct parameter estimates are selected in the model identification. On the other hand, in the model estimation procedure, regression weights of each measurement are determined before evaluating the goodness of fit (model testing step). Finally, the model would then be modified if the goodness of fit statistics do not indicate a good fit (model modification step), which is only performed in an Exploratory Factor Analysis (EFA). This study, however, was a Confirmatory Factor Analysis (CFA) which involves confirming an a priori model.

### Structural equations

The SEM procedure involves two equations, the measurement equation (observed) structural equation (unobserved). The observed indicators are measured in relation to the unobserved variables (latent). If a latent variable is predicted by another latent variables it is known as a ‘latent dependent’ variable or an ‘endogenous latent’ variable, and if a latent variable is not predicted by any latent variable it is called a ‘latent independent’ variable or an ‘exogenous latent’ variable. A latent variable can also be both independent and dependent and thus called a ‘mediating latent’ variable [18]. The structural relationships along the pathway of MNCH care in this study are presented in the following models:

1 $$ \mathrm{ANTENAT}={\beta}_1\mathrm{PERSONAL}+{\beta}_2\mathrm{COMMUN}+{\beta}_3\mathrm{S}\mathrm{E}\mathrm{S}+{\varepsilon}_{cov} $$

2 $$ \mathrm{DELIVERY}={\beta}_4\mathrm{PERSONAL}+{\beta}_5\mathrm{COMMUN}+{\beta}_6\mathrm{S}\mathrm{E}\mathrm{S}+{\beta}_7\mathrm{ANTENAT}+{\varepsilon}_{cov} $$

3 $$ \mathrm{POSTNAT}={\beta}_8\mathrm{PERSONAL} + {\beta}_9\mathrm{COMMUN}+{\beta}_{10}\mathrm{S}\mathrm{E}\mathrm{S}+{\beta}_{11}\mathrm{BABY}+{\beta}_{12}\mathrm{ANTENAT}+{\beta}_{13}\mathrm{DELIVERY}+{\varepsilon}_{cov} $$

4 $$ \mathrm{VACCINAT}={\beta}_{14}\mathrm{PERSONAL}+{\beta}_{15}\mathrm{COMMUN}+{\beta}_{16}\mathrm{S}\mathrm{E}\mathrm{S}+{\beta}_{17}\mathrm{BABY}+{\beta}_{18}\mathrm{ANTENAT}+{\beta}_{19}\mathrm{DELIVERY}+{\beta}_{20}\mathrm{POSTNAT}+{\varepsilon}_{cov} $$

### Model evaluation

Even though the conditions of the goodness-of-fit of a model differ among authors, the traditional method has been the absolute fit indices (e.g. *χ*^*2*^). However, since *χ*^*2*^ is sensitive to a large sample size and always rejects the proposed model [15], the Root-mean-square error of approximation (RMSEA) which is not sensitive to sample size is often considered [19]. Our study involved an extremely large sample size (*n* = 137,505), therefore, the traditional measure of RMSEA below 0.08 was used to indicate a good fit [20]. Other fit indices, such as model parsimony and incremental fit indices, are sometimes considered if one is performing an EFA which requires model modification [18].

### Ethical consideration

High ethical standards are always upheld in the collection, analysis and dissemination of the DHS data in SSA which are described in detail elsewhere [21]. Moreover, additional approval to use the data and to perform this particular study was also obtained from the DHS and the Institutional Review Board (IRB) of National Yang-Ming University (IRB: YM104021E).

## Results

### Geographical profile and inequality in MNCH care utilization

**Fig. 2:**
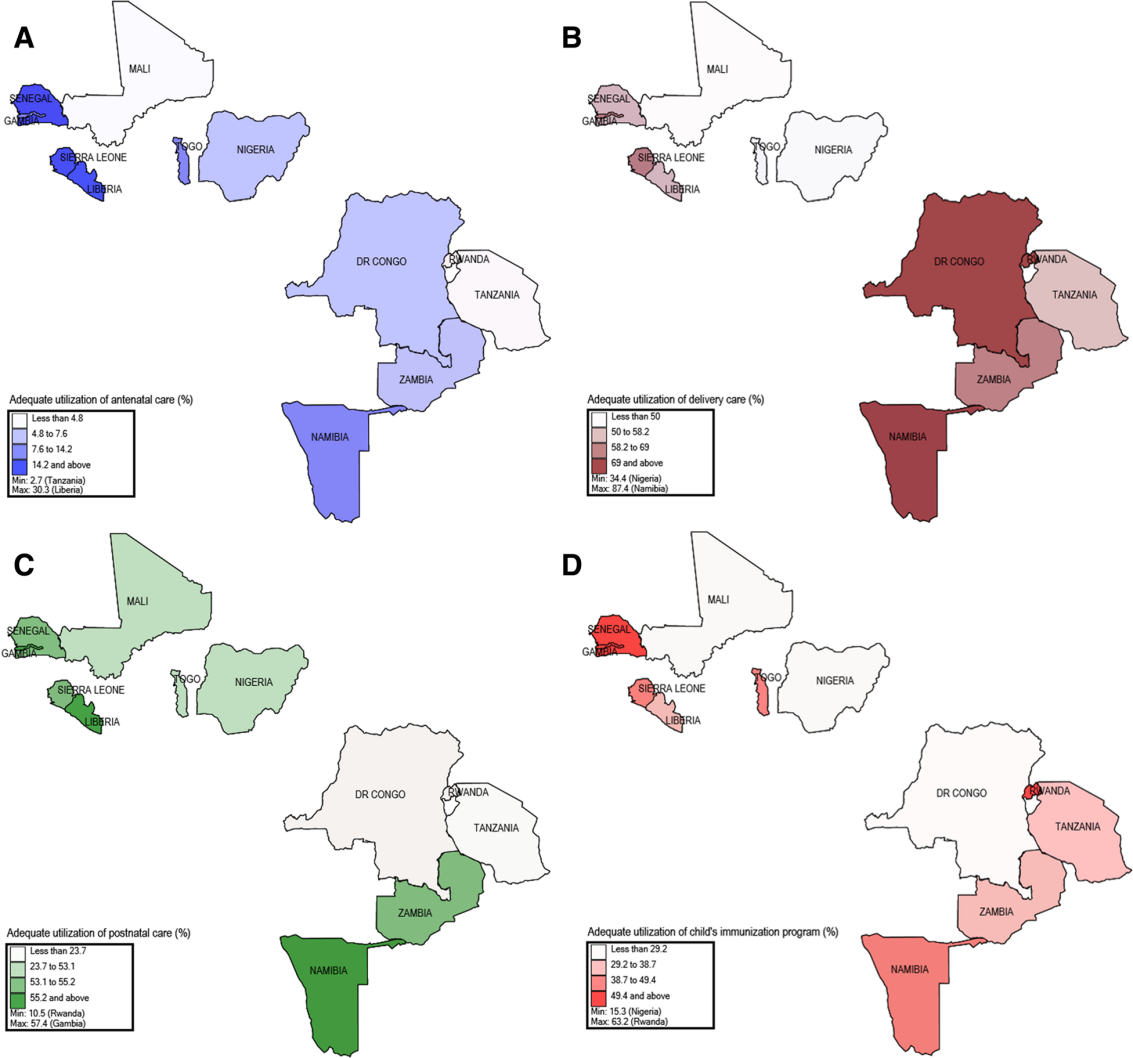
The distribution of the proportion of women who adequately utilized maternal, newborn and child health care in the 12 sub-Saharan Africa countries. **a** Adequate antenatal care, **b** adequate delivery care, **c** adequate postnatal care and **d** adequate child’s immunization

Figure 2 highlights the differences existing between countries by mapping out the national proportions of adequate utilization of MNCH care of the 12 countries. Some countries have a consistently low (Mali, Nigeria, DR Congo and Rwanda) or high (Namibia, Senegal, Gambia and Liberia) utilization in at least two levels of MNCH care. However, some countries may below in one level of MNCH care and stands out in another level of care (DR Congo and Rwanda). The differences in the adequate utilization of the four MNCH care services between the 12 countries were statistically significant (Table 1, *p* < 0.001). Table 1 shows that the majority of the respondents were married (88.2 %), living in rural areas (67.9 %), had large family (78.7 %), and without formal education (41.4 %). The proportions of women who utilized MNCH care and were without formal education were low in the four indicators than those who had secondary education and above. Mothers who were in the poorest quintile, not exposed to mass media, not employed and were married to fathers who were not employed and had lower education were less likely to utilize MNCH care adequately (Table 1).

**Table 1:** Proportion of women aged 15–49, who had a live birth in the 5 years preceding the survey, according to maternal and child health care utilization indicators in 12 sub-Saharan Africa countries

Characteristics	*N* = 137,505	Adequate antenatal care	Adequate delivery care	Adequate postnatal care	Adequate immunization
All	100	8.8	54.8	35.2	31.7
Country, year		***	***	***	***
DR Congo, 2013/14	13.6	4.8	78.3	20.2	20.5
Gambia, 2013	5.8	10.2	62.7	57.4	55.0
Liberia, 2013	4.8	30.3	55.1	55.2	31.4
Mali, 2012/13	7.7	4.7	39.2	27.5	18.7
Namibia, 2013	3.5	8.4	87.4	55.5	46.3
Nigeria, 2013	23.5	7.1	34.4	23.7	15.3
Rwanda, 2010	6.7	3.3	69.0	10.5	63.2
Senegal, 2013	4.6	22.6	58.2	54.2	40.1
Sierra Leone, 2013	9.0	14.2	54.0	54.2	49.4
Tanzania, 2010	6.0	2.7	50.0	17.5	35.9
Togo, 2013/14	4.9	7.6	45.0	50.1	38.7
Zambia, 2013/14	9.9	7.5	64.1	53.1	29.2
Marital status		***	***	***	***
Married	88.2	8.5	52.8	33.5	31.1
Formerly married	5.6	10.0	61.7	38.4	33.2
Unmarried	6.2	12.5	76.8	56.6	38.1
Age group		***	***	***	***
≤ 20	10.7	9.3	52.2	40.0	22.4
21–25	23.4	8.4	56.2	33.7	29.6
26–30	28.1	8.8	55.9	33.4	33.1
31–35	18.7	9.2	56.0	36.1	34.8
36–40	12.6	8.7	52.8	35.5	33.9
≥ 41	6.5	8.9	49.6	36.9	34.9
Birth order		***	***	***	***
1st	20.5	10.3	65.6	37.1	35.1
2nd	19.9	9.9	62.4	38.5	34.5
3rd–4th	27.5	9.1	52.7	34.4	31.0
> 4th	32.1	6.9	44.5	32.4	27.9
Family size		***	***	***	
≤ 6	21.3	10.3	60.2	37.4	31.9
> 6	78.7	8.4	53.3	34.6	31.6
Number of under-5, *M*		***	***	***	***
≤ 2	70.1	9.7	57.9	38.2	33.0
> 2	29.9	6.7	47.7	27.9	28.3
Residence		***	***	***	***
Urban	32.1	14.1	77.2	49.4	38.8
Rural	67.9	6.3	44.2	28.5	28.3
Mother’s education		***	***	***	***
No education	41.4	7.0	35.8	29.0	26.1
Primary	32.8	7.0	59.3	31.4	33.8
Secondary & higher	25.8	14.1	79.5	49.9	37.9
Wealth index		***	***	***	***
Poorest	22.7	4.7	34.8	24.0	24.1
Poor	22.2	5.9	41.7	28.6	26.7
Middle	20.3	8.3	52.5	34.8	30.9
Rich	18.7	10.7	70.2	42.8	36.4
Richest	16.1	17.1	86.1	51.8	44.7
Media exposure		***	***	***	***
Not exposed	31.2	5.2	41.9	24.5	21.0
Any one	32.7	7.8	49.7	33.2	34.0
Any two	26.6	11.4	65.7	43.9	36.7
All three	9.5	17.0	84.3	53.1	44.5
Mother’s employment		***	***	***	***
Unemployed	28.4	8.5	54.9	36.1	27.8
Professional	2.1	17.9	89.6	56.0	44.8
Middle level jobs	23.2	10.6	57.3	37.7	28.0
Agriculture	34.4	5.7	49.4	27.8	35.0
Other	11.9	11.3	59.1	43.0	35.9
Father’s employment		***	***	***	***
Unemployed	9.2	5.5	42.0	28.9	20.6
Professional	8.0	12.8	70.1	42.6	34.3
Middle level jobs	12.1	10.5	58.0	37.0	29.5
Agriculture	42.8	5.6	43.6	26.4	29.7
Other	27.9	11.9	64.6	42.0	36.3
Father’s education		***	***	***	***
No education	36.7	6.9	34.9	29.6	27.2
Primary	26.5	5.9	53.8	27.0	33.8
Secondary	30.1	10.4	69.4	40.0	31.7
Higher	6.8	18.7	78.6	51.7	39.1
Baby’s size		***	***	***	***
Very large	14.1	11.4	57.8	37.8	32.3
> Average	26.8	9.3	57.8	36.5	33.5
Average	43.3	8.1	54.6	34.9	31.8
< Average	10.8	8.1	52.7	33.7	30.3
Very small	5.0	9.8	50.4	35.6	28.2
Baby’s sex			**		
Male	50.5	8.9	55.3	35.2	31.7
Female	49.5	8.8	54.3	35.1	31.7
Baby’s age, years		***	***	***	***
< 1	21.2	12.2	57.8	50.1	9.0
1	20.3	12.3	56.9	50.4	44.2
2	19.4	10.0	56.2	38.6	43.3
3	19.8	6.2	53.4	24.0	39.3
4	19.3	4.3	52.5	17.2	36.6

**p <0.01; *** *p* < 0.001

### Model evaluation

**Fig. 3:**
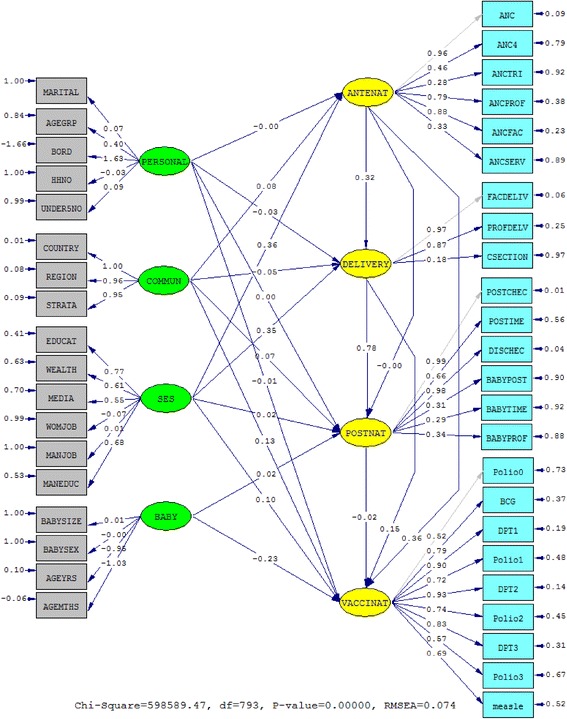
The standardized parameter estimates of the continuum of care for maternal, newborn and child health care of the 12 sub-Saharan Africa countries. The *oval-shaped* are latent variables and the *rectangle-shaped* are the measurements

Figure 3 indicates the generated model with the standardized parameter estimates. The global goodness-of-fit statistics produced in the FIML procedure shows a *χ*^2^ test statistic of 598,589.47, *df* = 793, *p* < 0.001 (normed *χ*^2^ = 3.42 or even 5.0); the large absolute fit index (*χ*^2^) is due to the large sample size in the study. However, the RMSEA which is not sensitive to the sample size indicated a good fit (RMSEA = 0.074) lower than the acceptable traditional level of 0.08.

#### Determinants of MNCH care utilization measurement indicators

All the measurement variables used as determinants of MNCH care utilization (individual and family, community, socioeconomic status and baby’s characteristics) had high loadings on the respective latent construct and were statistically significant at *p* < 0.001, except for child’s sex (Table 2). In the latent construct ‘individual and family characteristics’, only the indicator family size had a negative coefficient (−0.03) with the highest being the birth order measurement which was positive at 1.63. Moreover, only mother’s employment measurement in the latent construct ‘socioeconomic status’ was negative (−0.07). Age indicator was also negative with the measurement indicator ‘size at birth’ being the only positive (0.01) indicator in the latent construct ‘baby’s characteristics’.

**Table 2:** Parameter estimates of measurement variables of the continuum of care for maternal and newborn health care of 12 sub Saharan Africa countries (*n* = 137,505)

Code	Variable	Std. est.^a^	Std. err.	*p*-value
VACCINAT	ADEQUATE IMMUNIZATION			
Polio0	Polio 0	0.52^b^	-	-
BCG	BCG	0.79	0.002	<0.001
DPT1	DPT 1	0.90	0.002	<0.001
Polio1	Polio 1	0.72	0.001	<0.001
DPT2	DPT 2	0.93	0.002	<0.001
Polio2	Polio 2	0.74	0.002	<0.001
DPT3	DPT 3	0.83	0.002	<0.001
Polio3	Polio 3	0.57	0.002	<0.001
measle	Measles	0.69	0.002	<0.001
POSTNAT	ADEQUATE POSTNATAL CARE			
POSTCHEC	PNC after delivery	0.99^b^	-	-
POSTIME	PNC in 24 h after delivery	0.66	0.001	<0.001
DISCHEC	Checked before discharge	0.98	0.001	<0.001
BABYPOST	Newborn care after birth	0.31	0.002	<0.001
BABYTIME	Newborn care in 24 h after birth	0.29	0.001	<0.001
BABYPROF	Skilled newborn care after birth	0.34	0.002	<0.001
DELIVERY	ADEQUATE DELIVERY CARE			
FACDELIV	Delivered at the facility	0.97^b^	-	-
PROFDELV	Skilled birth attendance	0.87	0.001	<0.001
CSECTION	Caesarean section	0.18	0.001	<0.001
ANTENAT	ADEQUATE ANTENATAL CARE			
ANC	ANC contact	0.96^b^	-	-
ANC4	≥4 ANC visits	0.46	0.002	<0.001
ANCTRI	First trimester visit	0.28	0.002	<0.001
ANCPROF	Skilled ANC	0.79	0.001	<0.001
ANCFAC	Health facility-based ANC	0.88	0.001	<0.001
ANCSERV	Essential ANC services	0.33	0.001	<0.001
PERSONAL	INDIVIDUAL & FAMILY CHARACTERISTICS			
MARITAL	Marital status	0.07	0.001	<0.001
AGEGRP	Age group	0.40	0.009	<0.001
BORD	Birth order	1.63	0.03	<0.001
HHNO	Family size	−0.03	0.002	<0.001
UNDER5NO	No. of under-5 children	0.09	0.002	<0.001
COMMUN	COMMUNITY’S CHARACTERISTICS			
COUNTRY	Country	1.00	0.007	<0.001
REGION	Province	0.96	0.08	<0.001
STRATA	Residence	0.95	0.42	<0.001
SES	SOCIOECONOMIC STATUS			
EDUCAT	Mother’s education	0.77	0.002	<0.001
WEALTH	Wealth index	0.61	0.004	<0.001
MEDIA	Media exposure	0.55	0.003	<0.001
WOMJOB	Mother’s employment	−0.07	0.004	<0.001
MANJOB	Father’s employment	0.01	0.004	0.008
MANEDUC	Father’s education	0.68	0.003	<0.001
BABY	BABY’S CHARACTERISTICS			
BABYSIZE	Size at birth	0.01	0.003	<0.001
BABYSEX	Sex	−0.000	0.001	0.522
AGEYRS	Age in years	−0.95	0.003	<0.001
AGEMTHS	Age in months	−1.03	0.04	<0.001

^a^ The Full Information Maximum Likelihood estimation procedure took into account the missing at random (MAR) and missing completely at random (MCAR) data

^b^ Reference indicator (with a unit factor loading) to set the scale of each of the endogenous latent (ETA) variables of the model; Std. est., standardized estimate; Std. err., standard error

#### Continuum of care for MNCH measurement variables

Furthermore, all the measurement variables in the endogenous variables of the utilization of MNCH care (antenatal, delivery, postnatal and immunization) contributed considerably to the model and were statistically significant at *p* < 0.001. The parameter estimates of the indicators of the ‘adequate immunization’ latent were above 0.52, while the measurement ‘newborn care in 24 h after birth’ was the lowest estimate (0.29) in the ‘adequate postnatal care’ construct. The indicator caesarean section (0.18) and first trimester visit (0.28) had the lowest coefficient in the latent construct ‘adequate delivery care’ and ‘adequate antenatal care’, respectively (Table 2).

### Structural model analysis

**Fig. 4:**
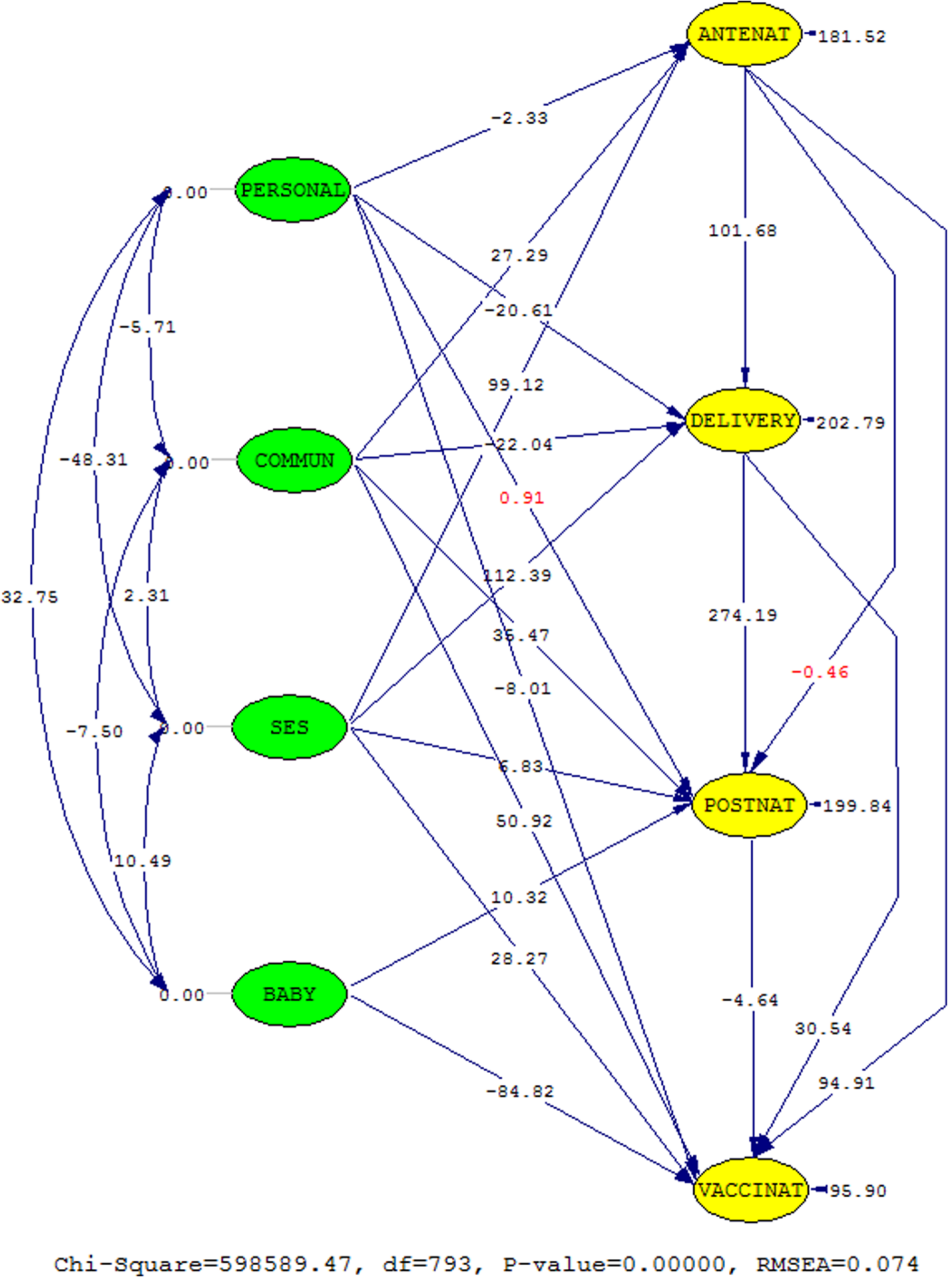
The *t*-values of the structural path relationships and the correlations of the independent latent variables. The values in *red* are not significant

Figure 4 shows the significant structural path relationships and exogenous variables’ correlations in the continuum of care for MNCH model of the 12 SSA countries. Only the path relationships from ‘adequate antenatal care’ to ‘adequate postnatal care’ (−0.001) and from ‘individual and family characteristics’ to ‘adequate postnatal care’ (0.001) were not significant at *p*-value 0.363 and 0.646, respectively (Table 3).

**Table 3:** Standardized parameter estimates of the structural equation model (Eq. 1, 2, 3 and 4)

Paths	Parameter	Std. Coeff.	Std. Error	*t*-value
PERSONAL → ANTENAT	*β*_1_	−0.004	0 · 002	−2.33*
COMMUN → ANTENAT	*β*_2_	0.08	0.003	27.29***
SES → ANTENAT	*Β*_3_	0.36	0 · 004	99.11***
PERSONAL → DELIVERY	*Β*_4_	−0.03	0.001	−20.61***
COMMUN → DELIVERY	*Β*_5_	−0.05	0.003	−22.04***
SES → DELIVERY	*Β*_6_	0.35	0.003	112.39***
**ANTENAT → DELIVERY**	*Β*_7_	0.32	0.003	101.68***
PERSONAL → POSTNAT	*Β*_8_	0.001	0.001	0.91
COMMUN → POSTNAT	*Β*_9_	0.07	0.002	35.47***
SES → POSTNAT	*Β*_10_	0.02	0.003	6.83***
BABY → POSTNAT	*Β*_11_	0.02	0.002	10.32***
**ANTENAT → POSTNAT**	*Β*_12_	−0.001	0.003	−0.46
**DELIVERY → POSTNAT**	*Β*_13_	0.78	0.003	274.19***
PERSONAL → VACCINAT	*Β*_14_	−0.01	0.001	−8.01***
COMMUN → VACCINAT	*Β*_15_	0.13	0.003	50.92***
SES → VACCINAT	*Β*_16_	0.10	0.004	28.27***
BABY → VACCINAT	*Β*_17_	−0.23	0.003	−84.82***
**ANTENAT → VACCINAT**	*Β*_18_	0.36	0.004	94.91***
**DELIVERY → VACCINAT**	*β*_19_	0.15	0.005	30.54***
**POSTNAT → VACCINAT**	*Β*_20_	−0.02	0.005	−4.64***

^a^ The Full Information Maximum Likelihood estimation procedure took into account the missing at random (MAR) and missing completely at random (MCAR) data; *Std. Coeff.* standardized coefficient, *Std. Error.* standard error. The continuum of care for MNCH paths are in bold

*p < 0 · 05, ***p < 0 · 001 (two-tailed)

#### Independent latent relationship to the continuum of care

The path relationships from ‘individual and family characteristics’ to ‘adequate antenatal care’ (−0.004), ‘adequate delivery care’ (−0.03) and ‘adequate child’s immunization’ (−0.01) were all negatively related were statistically significant. While, the path relationships from ‘community characteristics’ to ‘adequate antenatal care’ (0.08), ‘adequate postnatal care’ (0.07) and ‘adequate child’s immunization’ (0.13) were positive and statistically significant, only its relationship with ‘adequate delivery care’ (−0.05) was negative.

On the other hand, the latent construct ‘socioeconomic status’ had a positive relationship with all the four constructs in the continuum of MNCH care, with the highest estimate being in its relationship with ‘adequate antenatal care’ (0.36) and the lowest estimate being in the relationship with ‘adequate postnatal care’ (0.02). The ‘child’s characteristics’ latent had a positive relationship with ‘adequate postnatal care’ (0.02), but a strong negative relationship with ‘adequate child’s immunization’ (−0.23).

#### The continuum of care for MNCH

Only the path relationship between ‘adequate postnatal care’ and ‘adequate child’s immunization’ was negatively related (−0.02). All other relationships were positive and statistically significant, except the relationship between ‘adequate antenatal care’ and ‘adequate postnatal care’. The correlation coefficients of the exogenous constructs were all statistically significant as indicated in Fig. 4 (see also Table 4 in the Appendix for the covariance matrix of all the latent constructs and the correlation coefficients of the independent latent variables).

## Discussion

In the current setting, the growing awareness of the role of MNCH care in shaping equity in health and health outcomes of mothers and their babies, we explored distribution and relationships in the continuum of care for MNCH in some SSA countries. Even though most policies and programs focus on mothers and their children separately, they are interrelated in both health care needs and life. An understanding of how women utilize MNCH in most affected region (i.e. SSA), where mothers and their newborn die during or after birth is therefore essential [5].

Generally, in all the four MNCH care indicators used in this study, adequate utilization of antenatal care is lowest in the 12 countries (8.8 %) and adequate utilization of delivery care highest (54.8 %). The measures used in the pathway of care allowed for the distinction of countries that needs to enhance population coverage in two or more MNCH essential packages (DR Congo, Mali, Nigeria, Rwanda, Tanzania and Zambia) and those that would benefit from a more focused care (Gambia, Liberia, Namibia, Senegal, Sierra Leone and Togo). The countries that had high utilization of antenatal care subsequently experienced high utilization of other essential care along the pathway of MNCH care.

Maternal and infant mortality have declined since the last DHS, and yet, the proportions still remain high in SSA as compared to the South Asia and high-income countries [1, 2]. This is due to various socio-demographic, economic and health system related factors affecting utilization of MNCH care services negatively. Several studies have explored these factors [10, 12, 22, 23]. However, in this study we explored the exogenous effects of these factors on utilization of MNCH care i.e. the latent constructs. Our finding suggested that individual and family characteristics (PERSONAL) are negatively associated with utilization of antenatal care, delivery care and child’s immunization program in the 12 SSA countries. This finding is significant because families are considered vital in reducing maternal and infant mortality, since decisions to utilize any king of health care is done at the family level, that is—either to utilize the services of a traditional birth attendant or a skilled professional; and if individuals are not well informed or empowered to make appropriate choices, an increase death of both mothers and their newborns may still be realized in the post-MDGs. Our study finding also indicated that an increase in the proportion of mothers who utilized delivery and postnatal care as compared to those who utilized antenatal care, changed only the magnitude of the effect and not the direction of the estimate of the latent construct ‘individual and family characteristics.’

Moreover, we also found that the latent construct ‘community characteristics’ (COMMUN) was positively associated with antenatal care, postnatal care and immunization program but was negatively associated with delivery care. The role played by each country’s political structure, health system and each community towards encouraging utilization of essential services is important towards reducing maternal and newborn deaths in SSA. Figure 2 shows some countries that are relatively trying to encourage adequate utilization of MNCH such as Namibia, Gambia, Senegal and Sierra Leone and those that need to do more in some areas such as Mali, Nigeria, DR Congo, Tanzania and Rwanda.

The SEM analysis identified already known issue that socioeconomic status is a major determinant of utilization of care in SSA. We found that socioeconomic status had a strong positive relationship with all MNCH care constructs along the pathway of care. In SSA, most maternal and newborn deaths take place at home because of several barriers which impedes women’s choices, and hence, opting for a traditional birth attendant who cannot handle complications. Some of these barriers include extensive distances, monetary concerns, poor referral system between communities and hospitals. Our findings suggested that wealth, exposure to mass media and education of both parents were positively related with the use of MNCH care. The low literacy levels among poor families and inadequate health knowledge and services provided are key towards improving MNCH care utilization. Even though diverse measures of economic inequity have constantly been revealed in literature, each country needs a standard measure to target disadvantaged families and communities to encourage and enable mothers to utilize MNCH services adequately. An important obstacle in tackling this problem may be in the identification of the economically disadvantaged mothers who deserve special consideration.

Of worth noting is the finding that adequate utilization of antenatal care (ANTENAT) is directly and positively related with adequate utilization of delivery care and child’s immunization program (VACCINAT) but not utilization of postnatal care. The lack of the association between antenatal care and postnatal care was due to the sudden drop-out of women from the pathway of care between delivery care and postnatal care (34.2 %), hence some women who adequately utilized antenatal care services did not continued to postnatal care (23.8 %). A study indicated that while women who had at least one antenatal contact in SSA is fairly high (72 %) than in South Asia (68 %), the proportion of those who attain the recommended four antenatal visits is still low (42 %) [24]. Antenatal or delivery care is more than often sought for by women than postnatal care. Our study indicated that adequate utilization of antenatal care was lower than adequate utilization of postnatal care. However, this may not depict the reality since mothers who benefit from professionally supervised immediate postpartum care (which may be limited to a single check) are often neglected in the successive days and weeks. Utilization of care in each level of MNCH care therefore presents an opportunity for promoting a continued use of care along the pathway of care.

This study also found that adequate utilization of delivery care (DELIVERY) was positively associated with utilization of postnatal care and child’s immunization program. Even though skilled birth attendance is still low in SSA (45 %), no substitute currently exists [24]. On the other hand, adequate utilization of postnatal care (POSTNAT) was negatively related to child’s immunization program. The inability of mothers to effectively transition from delivery services and to later continue with care provided for their children is a major concern in SSA. The numerous obstacles to a facility-based care for mothers and their children, including the costs involved or transportation difficulties, calls for outreach visits and community-based care which can play a vital role in the development of the newborn and the health of the mother, regardless of where the birth took place.

### Policy and research implication

This is the first study, to our knowledge, that investigated relationships in the continuum of care for MNCH in SSA. In these countries where maternal and infant mortality is high, an understanding of how women utilize care can help in designing, implementing and prioritizing interventions aiming at improving MNCH and subsequently reducing pregnancy-, child birth- and post-delivery-related deaths. Individuals and community economic empowerment, for example, through community loans, would be pertinent in countries where household wealth has a major and direct effect to the utilization of MNCH care. But in countries where mothers’ and fathers’ education contributes to underutilization of MNCH care, the priority could be health promotion and education. The significant impact of the country’s health system and region of residence, free of the other determinants, reveals disparity in health care utilization across different communities and the difference within each country can be investigated further. Family and public participation in MNCH care is important, especially in SSA where a number of births and deaths occur at home. The role of MNCH care would be necessary towards achieving better health outcomes, even in the post-MDGs; and studies on the relationship between the continuum of care for MNCH and the health outcomes are therefore of great importance. To achieve the continuum of care, promotion of the next level of care should be sustained at each levels of care.

Our study has a number of potential limitations and strengths. First, the cross-sectional design of the study does not allow for establishment a causal relationship, thus a prospective study may be necessary in future. Second, information was collected retrospectively and relying on the mother’s will and ability to communicate the information and hence recall or self-reporting bias may also explain the associations in this study. Third, the DHS data was collected for general purposes and we were unable to control for the quality of data. Fourth, other determinants of utilization of MNCH care, for example, health-related problems were not included in the model. The interpretation of global patterns of the merged the data must be done paying attention to the diverse socioeconomic and political context the countries. However, several strengths in our study include: First, being the first study to examine the latent associations between different characteristics and the continuum of care for MNCH in SSA. Second, the use of a standardized and validated questionnaire and the representativeness of the study subjects give it strength. Finally, the method used to estimate the relationships in continuum of care for MNCH and the potential confounders we controlled for give it greater internal reliability.

## Conclusions

In conclusion, utilization of each level of care in the continuum of care for MNCH determines whether the next level of care will be utilized, that is—antenatal care is related to delivery care which is then related to postnatal and subsequently to child’s immunization program. The main determinants of utilization of all the levels of the continuum of care for MNCH were socioeconomic status and community’s characteristics. At the national level, identification of communities which are greatly contributing to overall disparity in health and a well laid out follow-up mechanism from pregnancy through to child’s immunization program could serve towards improving maternal and infant health outcomes and equity.

## Consent

High ethical standards are always upheld in the collection, analysis and dissemination of the DHS data in SSA which are described in detail elsewhere [21]. All women signed a written informed consent while parents of mothers aged between 15 to 17 years signed on their behalf.

### Availability of data and materials

The datasets supporting the conclusions of this article are available in the Demographic and Health Surveys program repository, [unique persistent identifier and hyperlink to datasets in http://dhsprogram.com].

## Appendix

**Table 4:** The covariance and correlation matrices of the latent constructs

	The latent variables covariance and correlation matrices							
	Covariance Matrix of all Latent Variables							
	ANTENAT	DELIVERY	POSTNAT	VACCINAT	PERSONAL	COMMUN	SES	BABY
ANTENAT	0.98							
DELIVERY	0.44	0.99						
POSTNAT	0.36	0.79	1.02					
VACCINAT	0.46	0.34	0.27	0.99				
PERSONAL	−0.06	−0.10	−0.08	−0.08	1.00			
COMMUN	0.09	−0.02	0.05	0.17	−0.01	1.00		
SES	0.35	0.47	0.39	0.29	−0.16	0.01	1.00	
BABY	0.01	0.01	0.03	−0.23	0.06	−0.02	0.03	1.00
	Correlation (SD) Matrix of Independent Variables							
	PERSONAL	COMMUN	SES	BABY				
PERSONAL	1.00							
COMMUN	−0.01 (0.001)***	1.00						
SES	−0.16 (0.001)***	0.01 (0.001)*	1.00					
BABY	0.06 (0.001)***	−0.02 (0.00)***	0.03 (0.00)***					

**p* < 0.05, ****p* < 0.001 (two-tailed)
