# Erratum to: Associations in the continuum of care for maternal, newborn and child health: a population-based study of 12 sub-Saharan Africa countries

**DOI:** 10.1186/s12889-016-3739-9

**Published:** 2016-10-10

**Authors:** Patrick Opiyo Owili, Miriam Adoyo Muga, Yiing-Jenq Chou, Yi-Hsin Elsa Hsu, Nicole Huang, Li-Yin Chien

**Affiliations:** 1 International Health Program, Institute of Public Health, School of Medicine, National Yang-Ming University, Taipei, Taiwan; 2 Institute of Community Health and Development, Great Lakes University of Kisumu, Kisumu, Kenya; 3 Institute of Public Health, School of Medicine, National Yang-Ming University, Taipei, Taiwan; 4 School of Health Care Administration, Taipei Medical University, Taipei, Taiwan; 5 Institute of Hospital and Health Care Administration, National Yang-Ming University, Taipei, Taiwan; 6 Institute of Clinical and Community Health Nursing, National Yang-Ming University, Taipei, Taiwan

## Erratum

Following the publication of this article [1] it was brought to our attention that funding information had failed to be included in the Declarations section of the article.

The authors would, therefore, like to add the following sentence: ‘This study was funded by National Science Council, Taiwan (grant number: 104-2314-B-010 -007 -MY3)’.
